# Biological Functions and Clinical Significance of the ABCG1 Transporter

**DOI:** 10.3390/biology14010008

**Published:** 2024-12-25

**Authors:** Stanislav Kotlyarov, Anna Kotlyarova

**Affiliations:** 1 Department of Nursing, Ryazan State Medical University, 390026 Ryazan, Russia; 2 Department of Pharmacy Management and Economics, Ryazan State Medical University, 390026 Ryazan, Russia; kaa.rz@yandex.ru

**Keywords:** ABC transporters, ABCG1, lipid metabolism, transport, cholesterol, atherosclerosis, reverse cholesterol transport, innate immune system

## Abstract

ATP-binding cassette (ABC) transporters are a large family of proteins that transport various substances across cell membranes using energy from ATP hydrolysis. ATP-binding cassette sub-family G member 1 (ABCG1) is a member of the ABCG subfamily of transporters and performs many important functions, such as the export of cholesterol and some other lipids across the membranes of various cells. Cholesterol transport is the mechanism that links metabolism and the innate immune system. Due to its lipid transport function, ABCG1 may contribute to the prevention of atherosclerosis and is involved in the functioning of the lung, pancreas, and other organs and systems. However, the full clinical significance of ABCG1 is still unknown and is a promising area for future research.

## Simple Summary

The current review describes the biological functions and potential clinical significance of the ABCG1 transporter. This protein is a member of the ABC transporter family, one of the best-known transport mechanisms in both prokaryotes and eukaryotes. ABCG1 is expressed in a variety of cells, where it can be found in both plasma and organelle membranes, through which it participates in cholesterol transport both in and out of the cell in a process known as reverse cholesterol transport. This function makes the ABCG1 transporter an interesting target for research.

## 1. Introduction

The active transport of chemically diverse substances across the plasma membrane of cells is one of the key characteristics of life. In this regard, ATP-binding cassette (ABC) transporters, which carry out the movement of various substrates against their chemical gradient across plasma membranes by utilizing energy from adenosine triphosphate (ATP) hydrolysis, play a very important role in the function of cells and the whole organism. ABC transporters are evolutionarily ancient proteins that are known in various living things ranging from prokaryotes to humans. In prokaryotes, ABC transporters can both export and import substances through the membrane, while in eukaryotes, they only export substances. ABC transporters are the main mechanism of cellular transport in bacteria [1], and in bacteria and archaea, about 1 to 3% of genomes encode ABC transporters [1,2,3]. Transported substrates in prokaryotes include a wide range of substances, including ions, amino acids, peptides, sugars, and other molecules. In Gram-negative bacteria, ABC transporters transport lipids and some polysaccharides from the cytoplasm to the periplasm. Exporters of both prokaryotes and eukaryotes function as pumps and are involved in the excretion of a wide range of substrates, including toxins and drugs, from the cell [4]. Among this group of exporters, the most studied ABC transporter is the P-glycoprotein (multidrug resistance 1 (MDR1)/ATP-binding cassette sub-family B member 1 (ABCB1)), as its functional activity in tumor cells underlies resistance to multiple drugs [5].

The export function of ABC transporters is explained by the “alternating access” model, which assumes switching between the inward-facing and outward-facing transmembrane cavities of the protein [6]. To move substrate, the dimerization of nucleotide-binding domains (NBDs) occurs, which induces a switching conformation of transmembrane domains (TMDs) that form a substrate-binding transport cavity [7]. This cavity alternately opens into either the cytoplasmic or outer leaflet of plasma membranes [8].

In humans, 48 ABC transporter genes have been identified, with ABC transporter proteins divided into seven subfamilies (ABCA-ABCG) based on their structural characteristics. At present, the role of several members of ABC transporters in the pathogenesis of various diseases is well described. Members of the ABCB, ABCC, and ABCG subfamilies are well known for their role in the mechanisms of multidrug resistance and are the subject of numerous studies on oncology. At the same time, the role of ABCA and ABCG subfamilies, including ABCG1, is known due to the participation of their representatives in lipid transport [2,5]. Although most ABCG members are associated with sterol transport, this is not their only known function. For example, ABCG2 (BCRP), a member of the ABCG subfamily, is also associated with multifactorial resistance to chemotherapy [9]. ABCG4, involved in cholesterol, desmosterol, and amyloid β-peptide transport, is also involved in doxorubicin-mediated chemoresistance [10,11]. Disorders of lipid homeostasis at both the cellular and organismal levels are of growing interest to clinicians and researchers. Indeed, new evidence suggests that lipid metabolism is closely linked to the innate immune system, and dysfunctions of the immune system underlie many chronic diseases. Thus, the aim of the current review is to discuss the biological function and clinical significance of the ABCG1 transporter.

## 2. Structure and Biological Functions of the ABCG1 Transporter

The *ABCG1* gene was originally identified as a homolog of the *Drosophila melanogaster* «white» gene, which acts as a dimeric importer of eye pigment precursors [12,13]. In humans, the *ABCG1* gene is located on the long arm of chromosome 21 (21q22.3) [2,12,14,15] and consists of 23 exons [16]. However, unlike its homolog, the protein encoded by this gene in humans is involved in the regulation of lipid metabolism, especially in the transport of cholesterol and phospholipids [13,17,18]. 

**Figure 1:**
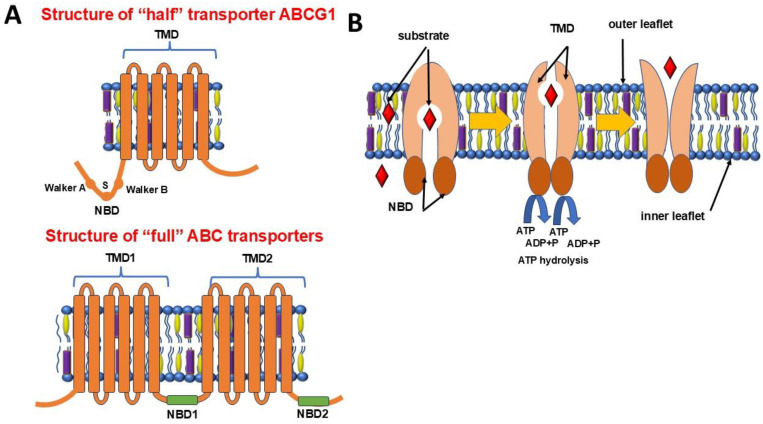
Structure of «half» transporter ABCG1 and «full» ABC transporters (**A**) and the ABCG1 transport function (**B**). Note: Unlike classical «full» ABC transporters, which consist of two repeating modules, ABCG1 is a «half» transporter containing one intracellular ATP-binding nucleotide-binding domain (NBD) and one transmembrane domain (TMD).

ABCG1 is characterized by a unique domain organization (Figure 1). Unlike classical «full» ABC transporters, which consist of two repeating modules, ABCG1 is a «half»-transporter containing one intracellular ATP-binding domain called the NBD at the N-terminus and one transmembrane domain. TMD ABCG1 consists of six transmembrane α-helices that form a pathway for transporting lipid molecules across the lipid bilayer. These helices contain hydrophobic amino acid residues that allow the interaction with membrane lipid components and substrates [18,19,20]. The NBD is located on the cytoplasmic side of the membrane and is responsible for ATP hydrolysis, providing an energetic contribution to the transport process. The NBD contains conserved domains (motifs), such as Walker A and Walker B, as well as an “S-motif” (Signature motif (S)) [16,21,22], which are required for the binding of the two NBDs and ATP and subsequent hydrolysis, where Walker A and Walker B stabilize this binding through a conserved amino acid residue in its structure [19,23].

Alternative splicing leads to the existence in humans of two protein isoforms, ABCG1(−12) and ABCG1(+12), (666 and 678 amino acids, respectively), which are distinguished by the presence of a 12 amino acid peptide (VKQTKRLKKGLRK) between the transmembrane and nucleotide-binding domains [16,24,25]. The functional activities of the two isoforms do not differ substantially between them, with the expression of the ABCG1(−12) isoform approximately twofold higher in human macrophages and vascular cells [16,24,25,26].

ABCG1 needs to form a dimer to function [16,17,27,28,29], and it forms most commonly a homodimer, but heterodimer formation with ABCG4 has also been described, for example in neuronal tissue, the eye, and induced macrophages [21,26,30,31,32,33,34,35,36,37,38]. The possibility of the heterodimerization of ABCG1 and ABCG4 is supported by the fact that the proteins share 72% common amino acid identity, but mainly ABCG1 and ABCG4 have different tissue distributions, making this possibility of heterodimerization a subject of research [21,26,31,39]. Dimerization results in the formation of a complete functional unit with two TMDs and two NBDs required for coordinated binding and the hydrolysis of ATP and the subsequent transport of substrates. ABCG1 dimerization occurs through interactions between the TMDs and NBDs of neighboring molecules. Hydrophobic and electrostatic interactions between domains ensure dimer stability. The results suggest that dimerization may be regulated by the lipid environment and the presence of substrates [19,26].

As already mentioned, the key function of ABCG1 is to provide an efflux of free cholesterol and phospholipids to the major high-density lipoprotein (HDL) fraction (HDL, HDL2, and HDL3), to pre-βHDL particles [33,40,41], but not to lipid-free ApoA-I [16,33,42,43,44,45], thus facilitating the removal of excess cholesterol in macrophages and peripheral tissues [20,26,33,46].

The process of substrate transport by the ABCG1 protein is a complex sequence of events involving coordinated conformational changes and the utilization of the hydrolysis energy of ATP, which is bound by the NBD (Figure 1) [16]. In this process, two ATP molecules bind to high-affinity sites in the NBD of each monomer [38]. ATP binding promotes a close interaction between the NBDs, forming a sandwich structure where ATP is positioned between the two NBDs. The dimerization of the NBDs induces allosteric changes in the TMD. The conformational changes cause the cytoplasmic cavity to close and the extracellular cavity to open, moving the substrate to the outside of the membrane. The substrate, most commonly cholesterol, is transported through a hydrophobic pathway in the TMD to the outer layer of the cell’s plasma membrane. After substrate transport, ATP hydrolysis to ADP and inorganic phosphate occurs. ATP hydrolysis reduces the affinity between NBDs, leading to their dissociation. Conformational changes after ATP hydrolysis promote substrate release on the outside of the membrane [38]. Cholesterol and phospholipids are transported to HDLs or apolipoproteins present in the extracellular environment, and the protein returns to its original state, ready for the next cycle of transport. It is hypothesized that, in the presence of inhibitors, the transporter is most likely blocked in the inward open conformation [38,47].

ABCG1 is expressed in many tissues and organs, including the kidney, liver, lung, brain, and spleen. High expression levels are observed in many cells, such as macrophages, lymphocytes, epithelial cells, and endothelial cells [16,20,22,30,33,36,42,43,46,48,49,50,51,52,53,54]. The cellular localization of ABCG1 is a matter of debate, as its localization both on the cell surface [30,33,46,53,54] and intracellularly has been reported, where a functional role of ABCG1 in the endosomal system has been described [2,13,16,21,23,34,43,45,46]. On CHO-K1 and HeLa cells overexpressing human ABCG1, it has been shown that the cytoskeleton plays a crucial role in the localization of ABCG1 at the plasma membrane [30]. Within cells, ABCG1 transports sterols by localizing in endocytic vesicles and promoting the redistribution of specific intracellular sterols from the endoplasmic reticulum [17,30]. Thus, ABCG1 may also be localized in the plasma membrane and endosomes [18,19,24,49,50,51,55,56]. ABCG1 is thought to circulate between intracellular and plasma membrane pools depending on the cholesterol level in the cell [30].

A growing body of evidence suggests an important role for ABCG1 in the function of various cells. ABCG1 is involved in T-cell proliferation [20,57] and has a protective role for apoptosis in macrophages [20,56,58]. In alveolar macrophages, ABCG1 is involved in maintaining the balance of the surfactant lipid composition necessary for normal lung function and regulates inflammatory responses in lung tissue by influencing macrophage activation [59,60,61]. In the liver, ABCG1 is involved in the regulation of cholesterol and phospholipids by promoting their efflux to HDLs and removes oxysterols and other potentially toxic lipid molecules from liver cells. In neurons and glial cells, ABCG1 is involved in the regulation of cholesterol levels important for synaptic plasticity and neuronal function, and in the blood–brain barrier cells it helps control lipid transport between the bloodstream and the brain [62]. ABCG1 also plays an important role in the vascular wall, where it is involved in maintaining endothelial integrity, regulating cholesterol levels and vascular reactivity, lipid homeostasis and inflammation in endothelial cells, in addition to being involved in reducing the effects of oxidative stress, which generally has an atheroprotective effect [63,64,65,66]. In macrophages, ABCG1 helps remove excess cholesterol in HDLs [52], preventing macrophages from turning into foam cells, a key step in the development of atherosclerosis. In addition, ABCG1 helps remove oxysterols and other potentially toxic molecules from macrophages [67,68]. Macrophage ABCG1 deficiency leads to significant lipid accumulation in macrophages and also influences the development of atherosclerotic lesions [60].

Among ABCG subfamily members, ABCG1 has some structural and functional similarities with ABCG4 and ABCG5/ABCG8, which include domain organization (since these proteins are semi-transporters and require dimerization), and substrate specificity, since they transport lipids and cholesterol [18,33]. However, these transporters have different expressions in tissues and organs and in regulation. Another member of the subfamily, ABCG2, is also involved in the export of lipids, such as some steroids and sphingosine-1-phosphate, but unlike ABCG1, its function is mainly related to the export of xenobiotics from cells, which is of clinical importance in oncology [69,70,71,72,73]. As mentioned above, ABCG1 and ABCG4 can form both homo- and heterodimers, ABCG2 forms only homodimers, and ABCG5 and ABCG8 are obligate heterodimers. On the other hand, in its key lipid transport function, ABCG1 is similar to a representative of another subfamily of ABC transporters: ABCA1. These transporters perform similar functions of cholesterol export and both are involved in the process of reverse cholesterol transport [65,74].

Thus, ABCG1 is a widely expressed protein in various organs and tissues that performs lipid transport functions in various cells. In this regard, there is growing research on and clinical interest in this transporter as a potential participant in metabolic and immune processes.

### 2.1. Participation of ABCG1 in Reverse Cholesterol Transport

Cholesterol is an important component of plasma cell membranes as it is essential for their structure and function. Cholesterol provides the biophysical properties of plasma membranes, such as viscosity and fluidity. The balance of cellular cholesterol content is provided by its synthesis, entry into the cell, and degradation and excretion pathways. The pathways of cholesterol biosynthesis and the mechanisms by which these pathways are regulated have been studied in considerable detail [75]. Reverse cholesterol transport is an equally important physiologic process by which excess cholesterol is excreted by HDLs from tissues to the liver. Reverse cholesterol transport has important clinical significance because it provides the physiologic regulation of tissue cholesterol homeostasis and protects the arterial wall from the development of atherosclerosis. ABCA1 and ABCG1 transporters are believed to play a key role in the process of reverse cholesterol transport [46]. It is known that ABCA1 participates in the formation of nascent HDLs promoting cholesterol outflow to lipid-free apolipoprotein A-1, and ABCG1 carries out the subsequent saturation of HDLs with cholesterol [76].

ABCG1 transporters are thought to not only transport cholesterol, but are also regulated by it. Proteins that interact with cholesterol are thought to have specific amino acid sequences in their transmembrane domain that are involved in this interaction. Such an amino acid sequence includes the cholesterol-binding domain (CRAC, Cholesterol Recognition/interaction Amino acid Consensus sequence) [77,78,79,80]. The CRAC domain includes the following set of amino acids: (L/V) − X(1-5) − (Y) − X(1-5) − (R/K). Another sequence is known as the CARC motif. It has similar binding properties to transmembrane proteins, but has the reverse amino acid sequence, i.e., (R/K) − X(1-5) − (Y/F) − X(1-5) − (L/V), where X is any amino acid. In this sequence, tyrosine can be replaced by phenylalanine [79,81,82,83]. ABCG1 has been shown to have a CRAC motif that allows the transporter to interact with cholesterol [78,84]. Structural analysis of ABCG1 showed that all CRAC/CARC motifs of ABCG1 in the transmembrane (TM) regions are clustered in TM6, a region that is essential for ABC transporter function and substrate recognition. The CRAC motif located around Y667 was found to represent a key cholesterol binding site, and this site is important for the transport function of ABCG1 [78]. It was also shown that the CRAC motif is important not only for functional activity, but also for protein stability by post-translational regulation. This is due to the fact that ABCG1 is ubiquitinated and degraded by the action of proteasome when the level of cholesterol in the cell decreases [85,86]. Thus, the structure of the ABCG1 transporter has close ties with cholesterol, which is not only its substrate for transport, but also potentially its regulator [78,86]. Thus, the function of ABCG1 in cholesterol transport is closely related to cholesterol itself, which is of great importance for its biological role.

### 2.2. The Role of ABCG1 in the Innate Immune System

The innate immune system is an evolutionarily ancient arm of immunity that provides nonspecific defense to the body against foreign pathological agents. The innate immune system relies on many evolutionarily conserved tools that are involved in agent detection and response. Macrophages are an important part of the innate immune system. These cells have different functions during inflammation. In a mouse model, it has been shown that macrophages can polarize into several phenotypes according to their role in inflammation. Proinflammatory M1 macrophages and M2 macrophages involved in the resolution of inflammation are known [87,88]. Although this classification is very simplified compared to the real picture, it is a convenient model for studying immunometabolic relationships, as the polarization of macrophages into these subtypes is associated with their metabolic reprogramming. ABCG1 is thought to be involved in macrophage polarization, as ABCG1 deficiency promotes increased cholesterol accumulation and polarization of macrophages into a proinflammatory M1 phenotype, which is mediated through the Akt signaling pathway and NF-κB activation [58]. In this case, ABCG1 deficiency corresponds to the decreased chemotaxis of M2 macrophages [89,90,91].

The proinflammatory role of ABCA1 and ABCG1 transporters in macrophages may be related to changes in the cellular content of cholesterol in macrophages. Cholesterol accumulation in plasma membranes is known to affect the function of membrane proteins, including mechanisms of direct cholesterol–protein interactions. Receptors of the innate immune system, such as Toll-like receptor 4 (TLR4), have membrane localization and may be regulated by membrane cholesterol content [82]. The genetic deficiency of ABCA1 and/or ABCG1 has previously been shown to promote increased lipid raft formation, increase TLR4 expression on the cell surface, and induce enhanced inflammatory responses in response to LPS [76,92,93,94,95,96,97]. In addition, cholesterol, which is an important component of the plasma membrane, can affect the clustering of TLRs, leading to the activation of downstream signaling pathways. Thus, the lipid composition of the plasma membrane has a significant impact on TLR4 structure and function, as the cross-talk between the innate immune system and cell plasma membrane structure is crucial for receptors of the innate immune system.

**Figure 2:**
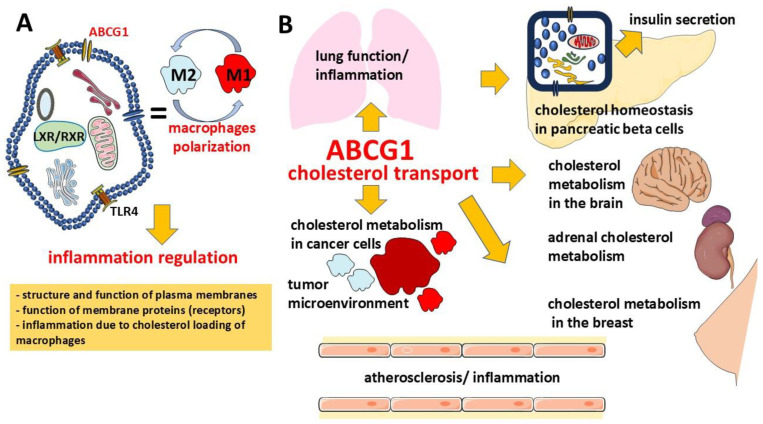
Biological and potential clinical significance of the ABCG1 transporter. Through the regulation of cholesterol transport, ABCG1 is involved in the regulation of inflammation involving macrophages (**A**), with potential clinical relevance to atherosclerosis, tumor progression, pancreatic, adrenal, brain, and breast function (**B**).

Thus, the lipid transport function of ABCG1 ensures its participation not only in metabolic but also in immune mechanisms, which is potentially of clinical interest (Figure 2).

## 3. Clinical Significance of ABCG1

### 3.1. The Role of ABCG1 in Atherosclerosis

Atherosclerosis is one of the key problems of modern society, as it underlies such socially significant diseases as coronary heart disease, cerebral stroke, and peripheral arterial disease [98,99]. Atherosclerosis is a process that develops in the vascular wall over many years as a result of a complex chain of events involving immune and metabolic mechanisms in which ABC transporters, including ABCG1, may be involved [100].

The *ABCG1* gene is known to contain a significant number of single-nucleotide substitutions, most of which are located in the regulatory regions of this gene. In this regard, it can be assumed that such structural changes may entail changes in the cholesterol transport function of the protein. For example, it was shown that ABCG1-mediated cholesterol efflux was reduced by 23% in the rs57137919 A/A genotype compared with the G/G genotype. In addition, the apoptosis of cholesterol-loaded macrophages was significantly increased in the A/A genotype compared with the G/G genotype. This was consistent with increased mRNA levels of the proapoptotic genes Bok and Bid in macrophages with the A/A genotype compared to macrophages with the G/G genotype [101]. In addition, *ABCG1* polymorphisms have been shown to be associated with the risk of atherosclerosis and its clinical manifestations. One study showed a significant association of genetic variants rs1378577 and rs1893590 of *ABCG1* with total plasma cholesterol concentration in residents of Northwestern Russia [102]. In another study, the rs57137919 locus genotype was shown to be associated with HDL cholesterol and low-density lipoprotein (LDL) cholesterol levels in Han Chinese [103]. The *ABCG1* polymorphism, g.-376C> T, resulted in a decreased ABCG1 mRNA expression, which increased the risks of CHD and myocardial infarction and resulted in shorter life expectancy in the general population. It has also been shown that a variation in the coding region of the *ABCG1* gene resulting in an amino acid substitution in ABCG1 (Ser630Leu) significantly increases the risks of myocardial infarction and coronary heart disease in general [104]. In a study on Japanese men, the rs5601744 polymorphism of the *ABCG1* promoter-(257T>G) was shown to influence the severity of CHD [105]. On the other hand, patients with the ABCG1-367G>A polymorphism (rs57137919) had a reduced risk of CHD and myocardial infarction (MI), and showed an association with CHD severity by angiography (multivessel CHD versus single-vessel CHD) [106]. Thus, changes in the structure of the ABCG1 transporter may be reflected in its function. Thus, it is of interest to study the relationship of *ABCG1* gene polymorphisms with clinical effects. At the same time, the mechanisms of how gene polymorphisms are related to protein transport function, substrate specificity, sensitivity to inducers or inhibitors, cell localization, protein stability, and posttranslational modification are still a subject of research.

A clinical evaluation of the significance of ABCG1 showed that the hypermethylation of the *ABCG1* gene was significantly associated with early signs of cardiovascular atherosclerosis (carotid intima-media thickness (cIMT)) [107]. At the same time, the hypermethylation status in the promoter region of the *ABCG1* gene was associated with ischemic stroke, and this association was more significant in women [107].

Thus, ABCG1, being involved in cholesterol transport, represents an interesting target for investigation in atherosclerosis.

#### 3.1.1. Function of ABCG1 in Macrophages in Atherosclerosis

Macrophages are key participants of atherogenesis. The population of these cells in the vascular wall in atherosclerosis is provided both by the influx of monocytes from the peripheral blood flow with subsequent differentiation into macrophages and from their own population of tissue macrophages. The evidence accumulated to date suggests that these cells play a differentiated role in inflammation in the vascular wall, demonstrating both pro- and anti-inflammatory phenotypes depending on their localization in the plaque, the activity of its progression, and instability [108,109]. Lipid overload of macrophages and foam cell formation are considered to be important stages in atherogenesis.

Given the contribution of ABCG1 to the process of reverse cholesterol transport in macrophages, its involvement in the protection against the development of atherosclerosis (Figure 2) is also suggested, and conversely the development of atherosclerosis is enhanced when the expression of this transporter is low. However, unlike ABCA1, the deficiency of which is associated with Tangier disease characterized by low HDL levels and early development of atherosclerosis [110], there are no data on a similar pattern for ABCG1 deficiency, which is probably compensated for by the ABCA1 function. Several studies have shown that lower levels of *ABCG1* mRNA and ABCG1 protein were found in macrophages from patients with atherosclerosis [111,112]. Moreover, patients with arterial occlusions had lower levels of *ABCG1* mRNA in monocytes than patients with evidence of less stenosis and controls. There was no correlation between the levels of total cholesterol and HDL in blood plasma and *ABCG1* gene expression [111].

The deletion of *Abcg1* in macrophages disturbs lipid homeostasis in alveolar macrophages and moderately affects the development of atherosclerotic lesions in LDL receptor-deficient mice [60]. Meanwhile, combined *Abca1* and *Abcg1* deficiency promotes fat cell accumulation and accelerates the development of atherosclerosis in mice. Specifically, bone marrow-recipient mice with *Abca1* and *Abcg1* knockout mice exhibited accelerated atherosclerosis and extensive infiltration of the myocardium and spleen with foamy cell macrophages [113]. In another study, the effect of *Abcg1* deficiency on the development of atherosclerotic lesions in LDL receptor-deficient mice was shown to be stage-dependent. In the early stages of atherogenesis, the absence of ABCG1 leads to increased lesions, whereas in the later stages, enhanced apoptosis and/or compensatory mechanisms lead to a slower progression of atherosclerotic lesions [114]. Thus, the role of ABCG1 in macrophages in atherosclerosis, in contrast to ABCA1, is still not sufficiently clear and requires new studies.

#### 3.1.2. Function of ABCG1 in Endothelial Cells in Atherosclerosis

The data on the role of ABCG1 in the function of endothelial cells are of interest. Endothelial cells cover the entire vascular bed with a monolayer, providing not only a barrier between the blood and tissues, but also performing a number of important vascular functions. They provide hemodynamic control by detecting changes in blood flow patterns and producing a number of bioactive substances, such as nitric oxide. In addition, endothelial cells regulate the permeability of the vascular wall to substances and cells, which is important for atherogenesis. The key stage of atherogenesis is endothelial dysfunction, which is characterized by the impaired production of bioactive substances, such as nitric oxide, by endothelial cells, characterized by an increased expression of leukocyte adhesion molecules, increased interactions of monocytes with the endothelium, and increased permeability of the endothelial monolayer for substances and cells [115,116,117,118].

ABCG1 is highly expressed in endothelial cells. The absence of ABCG1 in the endothelium results in a significant decrease in cholesterol efflux to HDLs. Although the total cholesterol content in the endothelium is not altered, there is an alteration in the intracellular distribution of neutral lipids in *Abcg1* knockout-mice endothelial cells. ABCG1 deficiency in endothelial cells was also found to promote endothelial activation and endothelial–monocyte interaction. Also, decreased ABCG1 expression in the endothelium promotes a proinflammatory endothelial phenotype. ABCG1 deficiency in aortic endothelial cells was found to activate endothelial interleukin (IL)6-IL6R-STAT3 (signal transducer and activator of transcription 3) signaling, thereby enhancing the monocyte–endothelium interaction and vascular inflammation. *Abcg1* knockout-mice endothelial cells also showed an increased surface expression of the adhesion molecules E-selectin and intercellular adhesion molecule 1 (ICAM-1), important for the regulation of monocyte adhesion to the endothelium. In addition, a significant increase in the secretion of keratinocyte-derived chemokine (murine analog of IL-8), IL6, and monocyte chemoattractant protein-1 (MCP-1) was observed in aortic endothelial cells from *Abcg1* knockout mice. These chemokines are involved in the recruitment of monocytes in the vessel wall, generally suggesting that the aortic endothelium of *Abcg1* knockout mice is activated to interact more rapidly with monocytes, which contributes to atherogenesis [64,119].

*Abcg1* knockout mice showed an accumulation of 7-ketocholesterol in aortic endothelial cells, which contributes to endothelial dysfunction, because 7-ketocholesterol accumulation leads to endothelial nitric oxide synthase (eNOS) dimer degradation and decreased eNOS activity [120]. Reduced eNOS activity has been shown in *Abcg1* knockout mice [120]. The use of siRNA, which resulted in a 70% reduction in *ABCG1* mRNA expression in human umbilical vein endothelial cells (HUVECs), reduced eNOS protein expression by 50% and nitric oxide (NO) activity by 30% compared with the normal controls. This was accompanied by a decrease in intracellular cholesterol efflux in HUVECs [121].

Thus, ABCG1 deficiency in aortic endothelial cells enhanced the monocyte–endothelium interaction and increased inflammation in the vascular wall, whereas the overexpression of ABCG1 in the endothelium markedly reduced plaque size progression and reduced vascular inflammation, consistent with a decrease in the number of macrophages and smooth muscle cells in the vascular wall [64,122].

Thus, the data available to date suggest that ABCG1 may play a positive role in the prevention of atherosclerosis, but its clinical significance is still unclear and requires new studies.

### 3.2. The Role of ABCG1 in Pancreatic Function and Diabetes Mellitus

Diabetes mellitus is a widespread metabolic disease characterized by insulin resistance and elevated blood glucose levels, which entails many biochemical abnormalities and clinical consequences, reduces the quality of life of patients, and worsens the prognosis [123]. In addition to impaired carbohydrate metabolism, diabetes mellitus also affects lipid metabolism, which increases the problem of the comorbid course of diabetes mellitus and atherosclerosis. In type 2 diabetes mellitus, there is often an increase in triglycerides and small, dense LDL particles, and a decrease in HDL levels.

Insulin production is an important function of the pancreas and is carried out by β-cells, which have a number of unique features and are located in the islets of Langerhans. It was found that 1 mm^3^ of rabbit pancreatic parenchyma contains 47.7 islets, which is 2.2% of its volume [124]. Under normal conditions, β-cells detect changes in plasma glucose concentrations and respond by releasing insulin [125].

A growing body of evidence suggests the importance of cholesterol in pancreatic β-cell function [125,126,127]. As mentioned in previous sections, cholesterol ensures optimal physicochemical characteristics and the normal functioning of plasma cell membranes, and this is important for insulin production and secretion by pancreatic β-cells. This is because lipid rafts in pancreatic β-cells are specifically enriched in ion channels and receptors, as well as proteins critical for coupling the detection of glucose levels with insulin secretion. Importantly, cholesterol is essential not only for plasma membrane function in β-cells, but also for proper secretory granule formation because it can influence the interaction of specific granule components with lipid rafts [125]. The fact is that insulin in β-cells is stored in granules that are surrounded by membranes containing significant amounts of cholesterol. Given that a β-cell normally contains about 9000 insulin granules, its total surface area may be much larger than that of the plasma membrane, and accordingly a larger pool of cellular cholesterol is contained in insulin granule membranes [124,128,129]. Due to this need for cholesterol, pancreatic β-cells can uptake it from plasma lipoproteins and also synthesize it independently via the mevalonate pathway. The endoplasmic reticulum, an organelle that is also associated with insulin biogenesis, is actively involved in this process [128]. However, when overloaded with cholesterol, the endoplasmic reticulum fails to redistribute it in the cell, which reduces the transport of proinsulin into the Golgi apparatus and decreases the ability of β-cells to produce, process, and store insulin. Excess cholesterol is delivered to insulin granules and accumulated by them. The accumulation of excess cholesterol in insulin granules leads to an increase in granule size and the impaired remodeling of their membranes [130]. The result is an accumulation of immature insulin granules, which contributes to the decreased ability of β-cells to secrete the required amount of insulin [130,131]. It has also been shown that not only an excess but also a deficiency of cholesterol can have a negative effect, since the optimal level of cholesterol in the membrane of secretory granules is high (40–50 mol%) [132]. In an experiment on rats, the administration of lovastatin, a member of the pharmaceutical group of statins that inhibit the cholesterol biosynthesis pathway via β-Hydroxy β-methylglutaryl-CoA (HMG-CoA) reductase, resulted in an increase in the size of secretory granules, a decrease in the number of dense nuclei accumulating insulin, a decrease in insulin content, and impaired regulated insulin secretion [132]. Another study also showed that the chronic inhibition of cholesterol biosynthesis regulates the functional activity of the voltage-gated calcium channel (CaV), insulin secretory granule mobilization, and membrane fusion, leading to impaired β-cell function, which may contribute to the development of type 2 diabetes [133].

Thus, the maintenance of optimal cholesterol balance is very important for β-cell function. As in other tissues, ABCA1 and ABCG1 are the main participants in cholesterol efflux in pancreatic β-cells [127]. ABCG1 has been found to be required for the stabilization of newly formed insulin granules against lysosomal degradation, and ABCA1, although involved in this process, is less involved than ABCG1 [128]. Both transporters are also required for optimal glucose-stimulated insulin secretion, probably due to their complementary functions. However, the depletion of ABCG1 significantly reduces the cholesterol content of granule membranes, making them unable to pack insulin molecules, thus reducing the total number of insulin granules [128]. ABCG1 expression has been found to be decreased in type 2 diabetes [134]. Experiments on the *Abcg1* knockout mouse model showed impaired glucose tolerance and insulin secretion with normal insulin sensitivity [135].

Given these data, it can be observed that ABCG1 plays a potentially significant role in the development of diabetes mellitus, and its assessment may be clinically useful (Figure 2). A number of studies have shown that *ABCG1* methylation levels are associated with type 2 diabetes mellitus [136,137,138]. It has been shown that, for example, DNA methylation levels at the CpG13 and CpG14 *ABCG1* loci and increased methylation of the CpG15 locus are positively associated with the risk of developing type 2 diabetes mellitus [139]. These data suggest the epigenetic regulation of glucose metabolism and risk of type 2 diabetes mellitus.

Thus, cholesterol metabolism in pancreatic β-cells plays an important role in their function, which is of clinical importance. ABCG1, being a key participant of cholesterol transport in these cells, may play a potentially important clinical role, which requires further studies.

### 3.3. The Importance of ABCG1 for the Respiratory System

The research findings in recent years have increased the understanding of the role of ABCG1 for the respiratory system (Figure 2). This is because lipid homeostasis, including the lipid composition of surfactants, is critical for the respiratory function of the lungs. This lipid balance is maintained by many cells, and cholesterol metabolism plays an important role. In addition, the lungs and airways are in daily contact with a vast number of pathogens that are in the inhaled air. This requires a complexly organized immune system that is provided by innate and adaptive mechanisms. Alveolar macrophages are important participants in the immune defense of the lungs. These cells have multiple immune and metabolic mechanisms, including participation in reverse cholesterol transport pathways involving ABCG1 [58,140].

ABCG1 is expressed in various cell types in the lungs, including alveolar macrophages, epithelial cells, and type II pneumocytes [113,128,129]. In mouse experiments, ABCG1 expression in macrophages has been shown to play a role in the regulation of lipid metabolism and lung inflammation. Conversely, the absence of ABCG1 promotes progressive chronic lung inflammation due to the dysregulation of intracellular cholesterol levels [97,141].

ABCG1 regulates pulmonary surfactant metabolism in mice and in humans. ABCG1-deficient mice have been shown to accumulate surfactant, lipid-laden macrophages and lamellar bodies of epithelial type 2 pneumocytes, B1-lymphocytes, and immunoglobulins [142]. It has been shown that, in pulmonary alveolar proteinosis, surfactant excretion is impaired and frothy, lipid-laden alveolar macrophages are found, and an increased content of cholesterol metabolites in the lungs is determined, which corresponds to a decrease in ABCG1 in macrophages [143].

It has been shown that *Abcg1* knockout-mouse macrophages have an increased ability to engulf apoptotic cells, accumulate lipids, and also become apoptotic [58]. Experimental studies on *Abcg1* knockout mice have shown that, by 6–8 months after birth, massive infiltrates consisting of lymphocytes and macrophages and including cholesterol crystals form in their lungs. In addition, an increased expression of various cytokines and cytokine receptors was observed in the lungs of these mice. In particular, *Abcg1* knockout macrophages were shown to produce the proinflammatory cytokines IL-6, IL-1β, IL-1α, and IL-12, but had low levels of production of anti-inflammatory IL-10. Elevated levels of matrix metalloproteinases (MMPs)-8 and -12, which are involved in airway remodeling, were also detected in the lungs of these mice [58,97,141]. Inflammation in the lungs of *Abcg1* knockout mice was found to develop secondary to lipid accumulation. The loss of liver X receptor (LXR) activity, which regulates ABCG1, also led to lipid accumulation and fibrosis in tissues, as well as the formation of cellular infiltrates from B- and T-lymphocytes in the subpleural zone [144].

ABCG1 is also thought to be involved in apoptosis of cells, including macrophages [111,120,126,127]. ABCG1 deficiency in alveolar macrophages has also been shown to contribute to granulomatous lung inflammation, which is enhanced by concomitant ABCA1 deficiency. Histologic examinations of *Abcg1* knockout and *Abca1/Abcg1* double-knockout animals revealed large pulmonary granulomas and increased expressions of CCL2 and osteopontin, which are involved in the formation of pulmonary granulomas, in bronchoalveolar lavage (BAL) cells [59]. ABCG1 deficiency has been shown to promote granuloma formation and fibrosis upon MWCNT administration, and to contribute to increased apoptosis and efferocytosis in BAL cells. An increased expression of transforming growth factor-beta (TGF-β) in the lung, as well as an increased expression of TGF-β-related signaling molecules, IL-13 and Smad-3, correspond to fibrosis [145]. Moreover, multi-walled carbon nanotubes (MWCNTs) induced stronger apoptosis in bronchoalveolar lavage cells in *Abcg1* knockout mice compared to wild-type mice. This may be due to the high caspase 8 activity and Fas expression in *Abcg1* knockout mice injected with MWCNT. In addition, BAL cells from *Abcg1* knockout mice injected with MWCNT were characterized by higher levels of efferocytosis markers, milk fat globule EGF factor 8 (MFG-E8), and integrin β3 [145].

Thus, ABCG1 plays an important biological role in lung function, but despite similar data suggesting the potential involvement of ABCG1 in the pathogenesis of lung disease in experimental animal models, the data on the clinical significance of ABCG1 are still scarce.

### 3.4. The Importance of ABCG1 for the Central Nervous System

Cholesterol is known to play an important role in the function of the central nervous system. Indeed, about 25% of all cholesterol in the body is located in the brain. Moreover, cholesterol in the brain is predominantly found in special myelin membranes and 30% is found in neurons and glia [146,147,148,149]. It is known that cholesterol from the peripheral bloodstream cannot enter the brain through the blood–brain barrier, so it is synthesized by brain cells [150,151]. Interestingly, neurons are dependent on cholesterol produced by astrocytes [150,152,153]. This is because neurons predominantly produce lanosterol, but these cells have insufficient levels of 24-dehudrocholesterol reductase (DHCR24) and lanosterol 14-alpha demethylase (CYP51) to convert lanosterol [152]. Given the importance of cholesterol to brain cells, ABCG1 has been shown to be expressed in a variety of cells in most parts of the brain, including neurons and astrocytes [35,154]. In porcine primary brain capillary endothelial cells (pBCECs), ABCG1 was found to be located in the apical and basolateral plasma membranes, as well as in early and late endosomes. In the same study, ABCG1 was shown to mediate cholesterol transport from pBCECs to HDL3 [62].

It was shown that the *Abcg1* gene knockout in mice exacerbated traumatic brain injury-induced pyroptosis, apoptosis, neuronal damage, brain swelling, neurologic impairment, and brain damage volume. The same study found that the cerebroprotective effect of Abcg1 in traumatic brain injuries was partially dependent on the activation of the RXRalpha/PPARgamma pathway [155]. The association of the *ABCG1* polymorphism with the risk of ischemic stroke in a Chinese population has also been shown. In particular, the rs1378577 TG and GG genotypes and the G allele were associated with a decreased risk of atherothrombotic stroke [156].

Given the importance of cholesterol, it has been suggested that disturbances in brain metabolism are significant for the development of a number of diseases, such as Alzheimer’s disease, Huntington’s disease, and Parkinson’s disease [153,157,158]. A possible link between ABCG1 and Alzheimer’s disease may be through its effects on lipid metabolism in the brain, as clinical studies show that ABCG1 is involved in impairing the cholesterol efflux capacity in cerebrospinal fluid in patients with Alzheimer’s disease [150]. In addition, ABCG1 plays a role in amyloid β generation through the modulation of membrane α-, β-, and γ-secretases activity [150,159,160]. However, the data on the clinical relevance of ABCG1 in Alzheimer’s disease are conflicting and require further studies [161]. Thus, the clinical data on the role of ABCG1 are still insufficient, which requires further studies.

### 3.5. The Significance of ABCG1 in Cancer

The interest in the role of ABCG1 in cancer has increased in recent years (Figure 2). The interest in ABCG1 in tumors is largely due to its role as a cholesterol transporter, as cholesterol plays an important role in carcinogenesis. Cholesterol metabolism is significantly altered in cancer. This applies to cholesterol metabolism both at the level of the whole organism and at the levels of tumor cells and tumor microenvironment cells [162,163,164,165,166].

Tumor cells regulate cholesterol balance to meet the metabolic needs for growth and proliferation. In addition, membrane cholesterol content is of importance to tumor cells. Cholesterol is known to be an important component of plasma membranes, where its physicochemical properties are essential for the biophysical properties of membranes, such as viscosity and fluidity. Cholesterol is involved in the formation of lipid rafts, i.e., cholesterol-containing, ordered, dynamic, signaling platforms that house many receptors. The cholesterol content of plasma membranes directly affects the stability of lipid rafts and the function of these receptors and their signaling pathways, including through cholesterol–protein interactions. Among other things, lipid rafts serve as a platform for the transduction of oncogenic signaling pathways. Another mechanism associated with changes in cholesterol metabolism in cancer is related to the use of cholesterol metabolism to regulate the function of cells in the tumor microenvironment (TME) [165,167,168,169].

By acting in TME cells, ABCG1 can have a significant effect on tumor progression. In immune cells, cholesterol metabolism is involved in the regulation of their function. Macrophages, analogous to inflammation, play a differential role in oncogenesis. While M1 macrophages exert a cytotoxic function against tumor cells, in contrast, M2 macrophages promote tumor cell growth and proliferation. Tumor-associated macrophages (TAMs), with predominant M2 phenotype promote tumor cell proliferation and angiogenesis, provide continuous matrix exchange, suppress adaptive immunity, and are associated with poor prognosis [170]. ABCG1 can induce a shift in the macrophage phenotype from M1 to M2 through cholesterol efflux [89]. It was shown that macrophages in tumors were devoid of cholesterol, although the lung tumor cells themselves were rich in cholesterol [171]. Genetic deletion of Abca1 and Abcg1 eliminates tumor-promoting TAMs and slows tumor progression [172]. *Abcg1* knockout mice were shown to suppress the growth of MB49 subcutaneous bladder carcinomas and B16 melanomas by modulating the function of macrophages in the tumor, resulting in increased longevity. One reason for the slowing of tumor growth in these mice is the switching of the macrophage phenotype from tumor growth-supporting M2 to tumor-fighting M1 macrophages among cells in the tumor microenvironment. Macrophages in *Abcg1* knockout mice have a predisposition to M1 polarization, which is characterized by increased NF-κB activation and direct cytotoxicity toward tumor cells in vitro [89]. Thus, acting through these and other mechanisms, cholesterol may modulate immune responses and promote the evasion of tumor cells from immune surveillance.

ABCG1 overexpression promotes tumor cell proliferation and enhances the expression of anti-apoptotic proteins BCL2 and MCL1 in HKULC4 lung cancer cells. In addition, ABCG1 decreases the expression of miR-29a, miR-29b, and miR-29c in HKULC4 cells. Thus, ABCG1 may function as a modulator of proliferation, migration, invasion, apoptosis, and miRNA regulation in lung cancer cells [173]. Therefore, it is suggested that ABCG1 could be considered as a new potential diagnostic and therapeutic target in prognosis assessments [174,175,176].

Thus, the role of ABCG1 as a potential diagnostic and therapeutic target in cancer is of clinical interest and requires new studies to better understand the putative links of transporter function to tumor development and progression.

### 3.6. The Role of ABCG1 in the Adrenal Glands

Steroid hormones synthesized by the adrenal glands perform many important physiological functions, controlling metabolism and stress response, puberty and reproduction, blood pressure regulation, etc. Cholesterol is a steroid precursor and a key modulator of steroidogenesis. Despite the involvement of ABCG1 in the regulation of cholesterol balance (Figure 2), and consequently its potential involvement in the regulation of glucocorticoid production in the adrenal cortex, the role of the transporter in the function of this organ is not yet fully known. In a recent study, it was shown that the inactivation of ABCG1 in the adrenal cortex leads to an increased expression of genes that contribute to cholesterol availability through uptake and biosynthesis, as well as increased glucocorticoid production. *Abcg1* conditional knockout mice had a 74% increase in adrenal steroid production, including pregnenolone, progesterone, 11-deoxycorticosterone, corticosterone, and aldosterone [177]. On the other hand, in another study, *Abcg1* gene knockout mice had a decreased ability to produce corticosterone in response to adrenocorticotropic hormone (ACTH) stimulation or starvation compared to wild-type mice [178]. Another study found an increased expression of ABCG1 in aged mice, while the expression of genes involved in cholesterol homeostasis or corticosteroid synthesis in the adrenal glands remained unchanged [179]. Thus, the data on the potential involvement of ABCG1 in steroidogenesis are of interest, but this issue is still a matter of debate and should be investigated in more detail.

### 3.7. The Role of ABCG1 in Mammary Gland Development and Function

A growing body of evidence suggests an important role of cholesterol in the structure and function of mammary epithelial cells, especially during their proliferation. In lactating mouse mammary gland tissues, ABCG1 localization was detected predominantly in the glandular epithelium and in mammary adipocytes [180]. In this regard, ABCG1 is thought to contribute to the normal physiologic development of the mammary gland and is also involved in the transfer of cholesterol to milk composition (Figure 2). ABCG1 has been detected in milk fat globule (MFG) membranes, indicating its possible involvement in cholesterol exchange between mammary epithelial cells (MECs) and alveolar milk [181]. It is thought that ABCG1 together with the ABCA1 transporter may be involved in cholesterol exchange between MECs and neighboring cells (e.g., mammary adipocytes or stromal cells in the mammary gland). The presence of ABCA1 and ABCG1 within the MFG emphasizes their physiological importance in the processes of cholesterol secretion into milk. In addition, the detection of these proteins in both the basal and apical parts of the cytoplasm suggests that they provide a common mechanism for maintaining cholesterol homeostasis and coordinate cholesterol exchange between epithelial cells and the external environment, including the lumen of alveoli [181]. On the other hand, it has been shown that ABCG1 mRNA levels did not differ in mammary tissue during lactation and the dry period in dairy cows [182].

In addition, ABCG1 may play a role in breast cancer due to its role in cholesterol transport [183]. In an experiment on MCF-7 breast cancer cells, it was shown that the activation of LXR led to an increase in *ABCG1* genes and transporter proteins, which enhanced cholesterol efflux into isolated high-density lipoproteins (HDLs) and led to a decrease in intracellular and membrane-bound cholesterol levels required for tumor cell growth. Cholesterol reduction resulted in the suppression of cell proliferation and stimulation of apoptosis. Thus, ABCG1-dependent cholesterol efflux results in an antiproliferative effect on breast cancer [184]. However, another study showed no significant differences between LXR-β and ABCG1 expression levels in triple-negative breast cancer (TNBC) tissues compared with benign breast tissues [185]. Thus, ABCG1 may be involved in breast cancer through the regulation of cholesterol transport, but this role requires new research. In general, the contribution of ABC transporters to carcinogenesis remains a subject of research. ABCG2 for example is known as the human breast cancer-resistance protein (BCRP) and is a marker of resistance to breast cancer chemotherapy.

Thus, the clinical significance of ABCG1 in the breast is not well understood, requiring new studies.

## 4. Conclusions

ABCG1, a member of a large family of ABC transporters, plays an important physiological role in cholesterol transport. Given the involvement of cholesterol in many structural and regulatory mechanisms, ABCG1 is of great biological importance and potential clinical interest. At the current time point, ABCG1 is known to be involved in the cross-talk of lipid transport, metabolism, and the innate immune system, with implications for the development of atherosclerosis, diabetes mellitus, and lung disease. At the same time, convincing evidence that indicated the real clinical significance of the transporter as a diagnostic marker or therapeutic target is still lacking. In this regard, ABCG1 and its role in lipid metabolism and the innate immune system is a potentially interesting and useful target for the research.

## Data Availability

The data are available from the corresponding author.
